# Mapping small-effect and linked quantitative trait loci for complex traits in backcross or DH populations via a multi-locus GWAS methodology

**DOI:** 10.1038/srep29951

**Published:** 2016-07-20

**Authors:** Shi-Bo Wang, Yang-Jun Wen, Wen-Long Ren, Yuan-Li Ni, Jin Zhang, Jian-Ying Feng, Yuan-Ming Zhang

**Affiliations:** 1 Statistical Genomics Lab, College of Plant Science and Technology, Huazhong Agricultural University, Wuhan 430070, China; 2 State Key Laboratory of Crop Genetics and Germplasm Enhancement, Nanjing Agricultural University, Nanjing 210095, China

## Abstract

Composite interval mapping (CIM) is the most widely-used method in linkage analysis. Its main feature is the ability to control genomic background effects via inclusion of co-factors in its genetic model. However, the result often depends on how the co-factors are selected, especially for small-effect and linked quantitative trait loci (QTL). To address this issue, here we proposed a new method under the framework of genome-wide association studies (GWAS). First, a single-locus random-SNP-effect mixed linear model method for GWAS was used to scan each putative QTL on the genome in backcross or doubled haploid populations. Here, controlling background via selecting markers in the CIM was replaced by estimating polygenic variance. Then, all the peaks in the negative logarithm P-value curve were selected as the positions of multiple putative QTL to be included in a multi-locus genetic model, and true QTL were automatically identified by empirical Bayes. This called genome-wide CIM (GCIM). A series of simulated and real datasets was used to validate the new method. As a result, the new method had higher power in QTL detection, greater accuracy in QTL effect estimation, and stronger robustness under various backgrounds as compared with the CIM and empirical Bayes methods.

Numerous methods and mating designs have been proposed since the first interval mapping procedure was developed by Lander & Botstein^1^. The composite interval mapping (CIM) procedure^2^,^3^ remains one of the most popular methods for quantitative trait locus (QTL) mapping due to its simplicity of single locus scanning and ability to control genetic background information. The inclusive composite interval mapping (ICIM) developed by Li *et al*.^4^ modified the CIM method by fitting a multiple regression model to estimate the co-factor effects and then adjusting the phenotypic value by the estimated effects of the co-factors before performing the usual interval mapping. The ICIM method is more robust than the original CIM because a more efficient co-factor selection process has been implemented.

Up to now, numerous QTL have been reported for complex traits in animals, plants and humans. Among these QTL, most have small effects on complex traits^5^, and some are closely linked^6^. Although QTL mapping has proven to be useful for detecting major QTL with relatively large effects, it may lack power in accurately modeling small-effect QTL^7^. Meanwhile, closely linked QTL might be mistakenly estimated as a single QTL with a larger effect at the wrong position if they have the same direction in effects, or they might be missed if their effects are in opposite directions^1^,^8^,^9^,^10^. Due to the difficulty in detection of small-effect and closely-linked QTL, the genetic foundations of most complex traits are not well understood. To address this issue, it is necessary to reconsider the model and improve the way that polygenic background is controlled.

Genome-wide association study (GWAS) has been widely used in human, animal and plant genetics^11^,^12^,^13^,^14^,^15^,^16^,^17^,^18^,^19^. The GWAS data often includes a large number of markers, making co-factor selection infeasible. Thus, polygenic effects are often fitted to a mixed linear model to capture the genomic background information^11^,^12^. This treatment can help us improve the methods of QTL mapping, and overcome the subjectivity nature of the CIM in co-factor selection^20^,^21^. However, it is still difficult to detect small-effect and closely-linked QTL.

In the usual GWAS, one marker is tested at a time and the entire genome is then scanned. In QTL mapping, each pseudo marker is tested at a time until all putative positions are scanned. The effect of the current marker has been treated as a fixed effect. However, treating QTL effect as random may have some advantages over treating it as fixed effect^21^,^22^. For example, the random effect treatment places a prior variance to shrink the estimated effect and such a shrinkage estimate of the QTL effects more stable than the least squares estimate when the genotypes are skewly distributed^19^.

Previous studies showed higher power from multi-locus QTL detection as compared with single-locus linkage analysis^22^,^23^,^24^ and the single-marker GWAS analysis^19^,^25^,^26^. To improve the power and accuracy in mapping small-effect and closely-linked QTL, the multi-locus model approach should be considered. In this study, therefore, a genome-wide composite interval mapping (GCIM) in backcross or DH was proposed under the framework of multi-locus GWAS of Wang *et al*.^19^. Not only can the new method solve the problem in co-factor selection, caused by a large number of markers, but also can overcome the shortcomings of the single-marker analysis. More importantly, GCIM can improve the power and accuracy in detection of small-effect and closely-linked QTL.

## Results

### Comparison of the GCIM under various QTL-effect models and K matrices

To optimize the GCIM, random- and fixed-effect models and the K matrices calculated respectively from whole and part markers were considered in this study. In the part-marker case, only the markers flanked by QTL under study were deleted. Thus, the performances for the above four GCIM methods were investigated. In other words, each sample in all the simulation experiments was analyzed by all the four methods. Results from the comparison of K matrices from the whole and part markers under random effect model showed that the higher power in QTL detection and greater accuracy in QTL effect estimation were observed from the whole-marker-derived K matrix than from the part-marker-derived K matrix (Tables 1 and S1 to S6). The above differences for power and accuracy under random effect model were slightly better than those under fixed effect model, which was further confirmed from the comparison between random- and fixed-QTL-effect models under K matrix from whole markers, although most of them were not significant (Table 1). Therefore, in the new GCIM method, QTL effect was viewed as random and K matrix was calculated from the whole markers.

### Power in QTL detection and accuracy in QTL-effect estimation for all the simulated QTL

To validate the new GCIM, a series of Monte Carlo simulation studies was carried out. In the first simulation experiment, only 20 QTL were simulated. Each sample was analyzed by the GCIM, CIM and empirical Bayes methods. As a result, the new method and empirical Bayes had 25.20% and 25.80% higher average power in the detection of QTL than the CIM, respectively (P-values: 2.09E-4 and 1.60E-4, respectively), while there was no significant difference between the new method and empirical Bayes (P-value = 0.4790) (Tables S4 and S8). When polygenic background 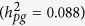 was added to the first simulation experiment and the polygenic background was changed into epistatic background 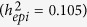, similar trends were observed (Tables S5, S6 and S8), although various P-values were observed.

We also used mean absolute deviation (MAD) to validate the new method. The new method and empirical Bayes had 0.44 and 0.49 lower average MAD in the estimation of QTL effect than the CIM, respectively (P-values: 0.0253 and 0.0162, respectively). When polygenic background 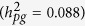 was added to the first simulation experiment and the polygenic background was changed into epistatic background 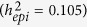, similar trends were observed (Tables S5, S6 and S8), although various P-values were observed.

As shown above, the contribution to increase the statistical power and to decrease the MAD varies from QTL to QTL. It is natural to make clear which kind of QTL has the maximum contribution to the increase of power and to the decrease of the MAD.

### Small-effect QTL

**Figure 1:**
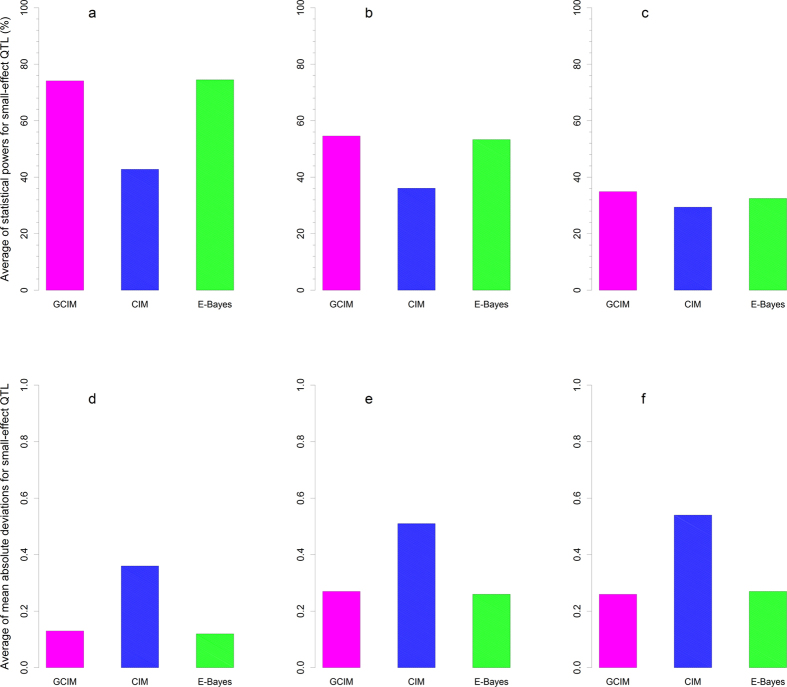
Average statistical power (**a–c**) and mean absolute deviation ((**d–f**), MAD) for small-effect QTL in the simulation experiments I (**a,d**), II (**b,e**) and III (**c,f**). The effects for the 9th, 14th, 19th and 20th QTL were small.

To make clear the reasons that result in significant difference in statistical power across various methods, we summarized the results from small-effect QTL. In this study, the 9th, 14th, 19th and 20th QTL were viewed as small, because their *r*^2^ were less than 1%. For these four small-effect QTL, the average powers for QTL detection from the new method and empirical Bayes had 31.38% and 31.75% higher than that from the CIM, respectively (P-values: 0.0178 and 0.0173, respectively), while no significant difference between the new method and empirical Bayes was observed (P-value = 0.7177) (Fig. 1, Tables S4 and S8). In the second and third simulation experiments, the same trends were found as well (Fig. 1; Tables S5, S6 and S8), although their P-values varied.

The accuracy for the QTL-effect estimate is also important for a new method. All the estimates from the three methods in the three simulation experiments were compared with their corresponding true values. MAD, MSE and SD were used to measure the accuracy. As a result, the new method and the empirical Bayes method had 0.23 and 0.24 significantly smaller average MAD for QTL effect than the CIM (P-values = 0.0115 and 0.0102) (Fig. 1, Tables S4 and S8). For other indicators and simulation experiments, the trends were similar except that the new and empirical Bayes methods were not statistically significant (P-values = 0.5456 and 0.5707) (Fig. 1; Tables S5, S6 and S8).

### Closely-linked QTL

**Figure 2:**
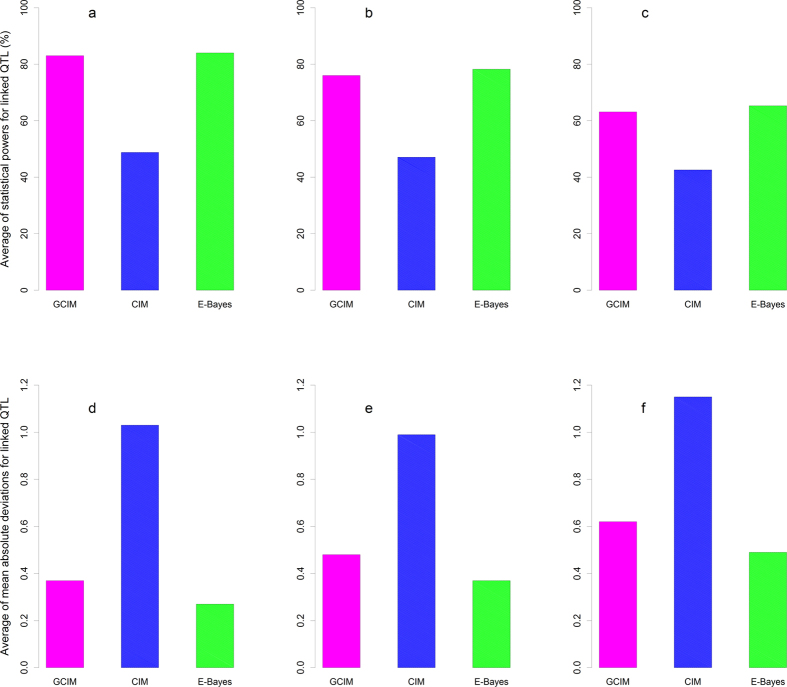
Average statistical power (**a–c**) and mean absolute deviation ((**d–f**), MAD) for closely-linked QTL in the simulation experiments I (**a,d**), II (**b,e**) and III (**c,f**). The 5th and 6th QTL, the 7th and 8th QTL, the 10th to 12th QTL, and the 16th to 18th QTL were closely linked.

To make clear the reasons that result in significant difference in statistical power across various methods, we summarized the results from closely linked QTL. If the map distance between two adjacent QTL is not larger than 20 cM, these QTL were viewed as linked QTL. For example, the 5th and 6th QTL, the 7th and 8th QTL, the 10th to 12th QTL, and the 16th to 18th QTL. For these ten closely-linked QTL, the statistical powers in the detection of QTL using the new and empirical Bayes methods were 34.25% and 35.20% higher than that using the CIM method, respectively (P-values: 4.92E-3 and 3.85E-3, respectively), while no significant difference between the new and empirical Bayes methods was observed (P-value = 0.5813) (Fig. 2, Tables S4 and S8). In the second and third simulation experiments, similar trends were found as well (Tables S5, S6 and S8), although their P-values varied.

### False positive rate

In the fourth simulation experiment, no QTL was simulated. At this case, all the QTL identified were false. If the number of QTL detected is large, the false positive rate is high. As a result, the number of QTL found in the fourth simulation experiment was 8, 160 and zero from the new method, CIM and empirical Bayes, indicating relatively low false positive rate from the new method.

### Real data analysis in triticale

To validate the new method, each of four crosses in Würschum *et al*.^27^ were analyzed for DS1, DS2 and DS3 by the CIM method, and all the four crosses were jointly analyzed for DS1, DS2 and DS3 by the new method, the empirical Bayes method and the method of Würschum *et al*.^27^. Würschum *et al*.^27^ detected 29, 14 and 14 QTL, respectively, for DS1, DS2 and DS3. All the results were listed in Tables 2 and S7.

The new method detected 27, 16 and 18 QTL, respectively, for DS1, DS2 and DS3. The corresponding numbers significantly associated with markers are 18, 10 and 12, respectively, from empirical Bayes. These significantly associated markers or QTL for each trait were used to conduct a multiple linear regression analysis and the corresponding Bayesian information criteria (BIC) were calculated. The new method shows the lowest BIC values for all the three traits (Table 3), indicating the best model fit from the new method.

In this study, two QTL detected by various methods are viewed to be similar if their positions are within 5 cM. As a result, there were 19, 11 and 10 similar QTL, respectively, for the above three traits, between the new method and the method of Würschum *et al*.^27^, 15, 14 and 11 similar QTL between the new method and the CIM method, and 14, 8 and 10 similar QTL between the new method and the empirical Bayes method. Clearly, these results validated the new method.

## Discussion

In the random model method of Wei & Xu^28^, the part-marker-derived K matrix method has higher power in QTL detection than the whole-marker-derived K matrix method. Similar results have been found as well in GWAS. The results are not seemingly consistent with our results in this study. Actually, they are identical, because smaller probabilities in the genome-wide scan (the first step) of the GCIM are found in the part-marker-derived K matrix case (data not shown). In the GCIM, only all the peaks in the genome-wide scan curve are selected as the positions of multiple putative QTL. The position changes for the putative QTL under the part-marker-derived K matrix may decrease the power and accuracy.

Although Xu^21^ used the random-QTL-effect mixed linear model framework of GWAS to identify QTL in backcross, his approach was not significantly better than the CIM in the Monte Carlo simulation experiments. The integration of multi-locus genetic model with Xu’s method^21^ in this study has significantly improved the statistical power of QTL detection. Although we adopted the GWAS methodology in this study, the new method is different from the GWAS methodology, because QTL mapping in backcross or DH populations can evaluate each possible genome position without marker. In this case, pseudo markers in every *d* cM need to be inserted in order to cover the entire genome, which makes the new method estimate QTL positions more accurately. This is another reason why the new method is called as GCIM, although the most important reason is that the new method may control polygenic background on a genome-wide level. Although the new method was proposed in backcross or DH, it is suitable for the mapping any populations with two genotypes, for example, recombinant inbred lines. The new method is also used to map QTL in chromosome segment substitution lines, but we can scan only marker positions, because conditional probabilities at the positions of pseudo markers can not be calculated. If the number of genotypes in a mapping population is more than two, for example, F_2_, the current method requires some modifications and further investigation will be conducted in the near future.

Here we compared the new method with the CIM, which is a widely-used QTL mapping method. The results from the new method showed higher power in QTL detection, higher accuracy in QTL effect estimation, and better model fit under various genetic backgrounds in the first to third simulation experiments, especially for small-effect and closely-linked QTL. The reasons are as follows. We scanned and selected markers with a low criterion of significance test. Potential QTL especially with small-effect or linkage cannot be excluded and can be easily included in the last model. In addition, we also compared the new method with inclusive CIM (ICIM) of Li *et al*.^4^. As a result, the new method has higher average power than the ICIM, especially for small-effect and closely-linked QTL (Table S9).

Although empirical Bayes is slightly better than the new method but no significant difference is observed in this study. Note that in this study empirical Bayes was implemented with multi-marker analysis. If pseudo markers were inserted between two adjacent markers and the effects for all the true and pseudo markers were simultaneously estimated by the empirical Bayes, the empirical Bayes was significantly worse than the new method (results not shown). If we increase marker density, the collinearity among closely linked markers will make parameter estimation of the empirical Bayes method difficult. If the number of the true and pseudo markers is many times larger than sample size, the empirical Bayes will fail. Thus, the new method is better than the empirical Bayes method.

## Conclusion

The mrMLM methodology for GWAS was used to conduct linkage analysis in backcross and DH populations. Genomic background can be effectively controlled by estimating polygenic variance. All the peaks of the negative logarithm P-value curve in genome-wide single-point scanning were selected as the positions of multiple putative QTL to be included in a multi-locus genetic model, and true QTL were automatically identified by empirical Bayes. The new method had higher power in QTL detection, greater accuracy in QTL effect estimation, and stronger robustness under various backgrounds as compared with the CIM and empirical Bayes methods, especially for small and closely linked QTL.

## Methods

The method is adopted from the mixed model genome-wide association studies^11^,^12^ where population structure and other non-genetic variables are treated as fixed effects and polygenes are fitted to the model as a random effect. The computational algorithm follows the eigen-decomposition approach used in the efficient mixed model association (EMMA) proposed by Kang *et al*.^14^ and in the improved genome-wide efficient mixed-model association (GEMMA) developed by Zhou & Stephens^18^. We propose to scan the genome by one marker at a time. The effect of the current marker is treated as a random effect^19^. In this study, when the marker effect is treated as random effect, the model is called the random effect model. For the paper to be self-contained, we briefly introduce both models in the next paragraphs.

### Genetic model

Let **y** be an *n* × 1 vector of phenotypic values for *n* individuals in a backcross or DH population. Let **Z**_*k*_ be a genotype indictor variable for marker *k*, where the *j*th element of **Z**_*k*_ is defined as


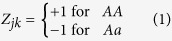


We now write the model by


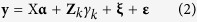


where **X** is a design matrix for (non-genetic) fixed effects **α**, *γ*_*k*_ is the effect of marker *k*. We now treat *γ*_*k*_ as a random effect with a 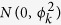 distribution. When treated as random, the estimated *γ*_*k*_ is a shrinkage estimator and also called empirical Bayes estimate because *ϕ*^2^ is also estimated from the data^29^. **ξ** is an *n* × 1 vector of polygenic effects and **ε** is the residual error. Assume that **ε** ∼ *N*(0, **I**_*n*_*σ*^2^) and **ξ** ∼ *N*(0, **K***ϕ*^2^), where *σ*^2^ is the residual error variance and *ϕ*^2^ is the polygenic variance. The covariance structure **K** is calculated using genome-wide marker information^21^.

QTL mapping differs from GWAS in that chromosome regions without markers also need to be evaluated. Essentially, we insert one pseudo marker in every *d* cM to cover the entire genome evenly so that every position of the genome will be evaluated. When a pseudo marker is located between two consecutive markers, we will use the multipoint method of Jiang & Zeng^30^ to calculate the genotype probabilities, denoted by *p*_*jk*_(*AA*) and *p*_*jk*_(*Aa*), respectively, for the two genotypes AA and Aa in backcross. The genotype indicator variable, *Z*_*jk*_, is then defined as the expected value conditional on flanking marker genotypes^31^. Therefore, *Z*_*jk*_ is defined as


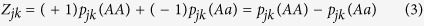


The expectation of **y** is E(**y**) = **Xα** and the variance is


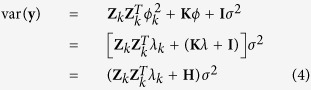


where 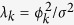 and **H** = **K***λ*+**I** where


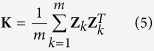


is a marker-inferred kinship matrix^21^.

### Parameter estimation

After absorbing *α* and *σ*^2^ we have two variance ratios to estimate for each marker, which are *λ*_*k*_ and *λ*. The fast algorithm of Zhou & Stephens^18^ does not apply to more than two variance components in its original form. The Newton-Raphson iteration algorithm must be used to search for the solution of two variance components. Here, we adopted the approximate approach implemented in EMMA^15^ and P3D^16^ to assume that the polygenic variance ratio is constant across loci and thus is replaced by the estimated value under the pure polygenic model (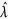) where no markers are fitted to the model. Once 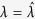 is fixed, we can still take advantage of the eigen-decomposition to speed up the genome scan process^19^.

Let y^*^ = **U**^*T*^**y**, X^*^ = **U**^*T*^**X** and 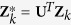 be transformed variables so that


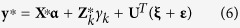


The variance-covariance matrix of y^*^ is


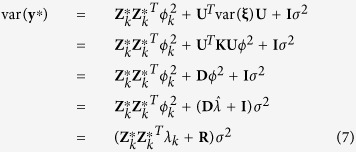


where 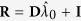 is a known diagonal matrix. Let 
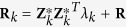
 be the general covariance structure. After absorbing *α* and *σ*^2^, we have the following profiled restricted log likelihood function,


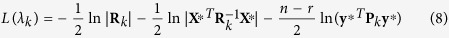


where


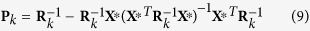


This likelihood function contains only one unknown parameter, *λ*_*k*_. The Newton algorithm for *λ*_*k*_ is


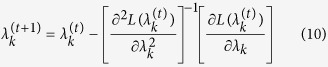


Once the iteration process converges, the solution is the REML estimate of *λ*_*k*_, denoted by 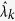. Given 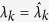, the estimates of *α* and *σ*^2^ are


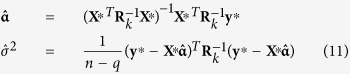


The best linear unbiased prediction (BLUP) of *γ*_*k*_ is also the conditional expectation of *γ*_*k*_ given y^*^ and has the following expression,


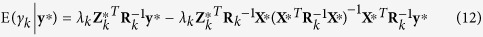


The conditional variance is


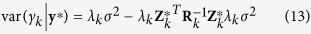


Under the random model approach, we first estimate the polygenic variance. We then estimate *λ*_*k*_ and test *λ*_*k*_ = 0 for each marker using the same estimated polygenic variance.

### Wald test for marker effect

We use the Wald test to test *H*_0_ : *γ*_*k*_ = 0 or *H*_0_ : *λ*_*k*_ = 0 in the random effect model approach, The Wald test statistic is


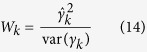


where 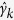 and var(*γ*_*k*_) are obtained from equations (12) and (13), respectively^32^.

### Multi-locus random-QTL-effect mixed linear model method

The single-marker random-QTL-effect mixed linear model (rMLM) method described above is considered as an initial scanning step for a new multi-locus random-QTL-effect mixed linear model (mrMLM) approach that is described here. In the rMLM step, the negative logarithm P-value curve is obtained for the whole genome. In the curve, all the peaks were selected as the positions of putative QTL in a multi-locus QTL mapping model. Thus, multi-locus genetic model under consideration is as follows


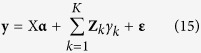


where *K* is the number of peaks in the negative logarithm P-value curve, **γ** = (*γ*_1_ *γ*_2_…*γ*_*K*_)^*T*^, and the others are same as those in model (2). Note that 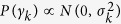 and other prior distributions are same as those in Xu^32^. In the above model, polygenic background is not included, because that all the potential QTL have been included in the model (15)^19^.

All the effects of QTL in the multi-locus model were estimated by empirical Bayes of Xu^32^. The procedure for parameter estimation is as follows.

(1) Setting initial values: 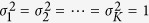, **α**=(**X**^*T*^**X**)^−1^**X**^*T*^**y** and 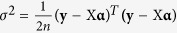;

(2) E-step: QTL effect can be predicted by


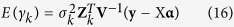


where 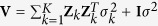;

(3) M-step: update parameters 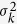, **α** and *σ*^2^


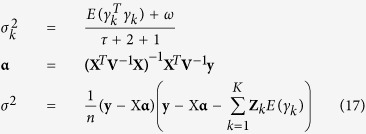


where 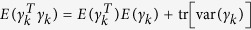 and 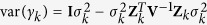. Repeat E-step and M-step until convergence is satisfied.

If the P-value for a random effect apart from zero was less than 0.01 in the F-test, the putative QTL were picked up to perform the likelihood ratio test (LRT). All the putative QTL with the ≥2.5 LOD score were viewed as true. Considering that all potential QTL were selected in the first stage, we decided to place a slightly more stringent criterion of 0.000691, which is converted from LOD score 2.50 of the test statistics using 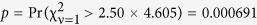.

In this study, the mrMLM method was used to detect QTL for the trait. The identified QTL were used to correct original phenotypes, and the corrected ones were analyzed again in order to increase the power.

### Real dataset analyzed

A large mapping population of triticale with 647 doubled haploid lines derived from four partially connected crosses (HeTi117-06 × Pawo, HeTi117-06 × TIW671, Modus × Saka3006 and Modus × Saka3008) was used for the demonstration^27^. All the plants were scored visually for their developmental stages at three time points termed DS1, DS2 and DS3, and scanned for genotypes. Only one marker was kept if several markers are located at the same position. All the 1549 markers with different positions covered 2,306.7 cM of the entire genome. The average marker distance was 1.5 cM. All the data was downloaded from http://www.g3journal.org/content/4/9/1585/suppl/DC1. We inserted one or more pseudo markers in intervals larger than 1 cM to make sure that the entire genome is evenly covered by pseudo or true markers with no intervals larger than 1 cM. The number of pseudo markers inserted was 1691, resulting in a total of 3240 markers. For the pseudo markers, the genotype indicator variable is missing for every individual. In this case, the missing variable was replaced by their conditional expectation.

### Monte Carlo simulation experiments

Each backcross population with 400 individuals was simulated. We placed one marker in every 5 cM and the entire chromosome was then evenly covered by 481 co-dominant markers. Twenty simulated QTL were located on a single large chromosome of 2400 cM in length. The effects and locations of the 20 QTL were listed in Table S1. These QTL vary in size with the largest QTL explaining 20% of the phenotypic variance and with the smallest QTL explaining 0.5% of the phenotypic variance. The population mean is 100 and the residual variance is 10. Each of the 200 simulated samples was analyzed by the new method, CIM and empirical Bayes. All the true and pseudo markers were scanned for the new and CIM methods but only the true markers were included in the full model for empirical Bayes. For each simulated QTL, we counted the samples in which the LOD statistic exceeded 2.5. A detected QTL within 5 cM of the simulated QTL was considered a true QTL. The ratio of the number of such samples to the total number of replicates (200) represented the empirical power of this QTL. The false positive rate (FPR) was calculated as the ratio of the number of false positive effects to the total number of zero effects considered in the full model. To measure the bias of QTL effect estimates, mean squared error (MSE) and mean absolute deviation (MAD),


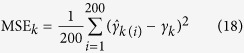



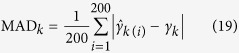


were calculated, where 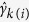 is the estimate of *γ*_*k*_ in the *i*th sample.

To investigate the effect of polygenic (small effect genes) background on the new method, polygenic effect was simulated by multivariate normal distribution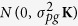, where 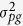 is polygenic variance, and **K** is kinship coefficient matrix between a pair of individuals. Here 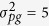, so 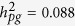. Other setups are identical to the first simulation experiment (Table S2).

To investigate the effect of epistatic background on the new method, three epistatic QTL pairs each with 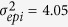 and 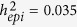 were simulated. The first one was placed between 800 cM and 1800 cM; the second one between 1210 cM and 1860 cM; and the last one between 275 cM and 740 cM. Other setups are identical to the first simulation experiment (Table S3).

To investigate the type I error for the new method, no QTL was simulated. We just simulated residual error in this simulation study. Other setups are identical to the first simulation experiment.

We developed our own software to implement all the analyses in this paper and would upload it to the R website (https://cran.r-project.org/web/packages/mrMLM/index.html).

## Additional Information

**How to cite this article**: Wang, S.-B. *et al*. Mapping small-effect and linked quantitative trait loci for complex traits in backcross or DH populations via a multi-locus GWAS methodology. *Sci. Rep*. **6**, 29951; doi: 10.1038/srep29951 (2016).

## Supplementary Material

Supplementary Information

Supplementary Dataset 1

## Figures and Tables

**Table 1:** Differences and their paired-*t*-test probabilities for average power and mean absolute deviation (MAD) obtained from genome-wide composite interval mapping in Monte Carlo simulation studies*.

Simulation experiment	K matrices from whole (*A*) and part (*B*) markers under random-QTL-effect model		K matrices from whole (*A*) and part (*B*) markers under fixed-QTL-effect model		Random- (*A*) and fixed-QTL-effect (*B*) models under K matrix from whole markers		Random- (*A*) and fixed-QTL-effect (*B*) models under K matrix from part markers	
	Power (%)	MAD	Power (%)	MAD	Power (%)	MAD	Power (%)	MAD
All the 20 QTL								
I	1.53 (0.0004)*	−0.025 (0.0069)	0.90 (0.0628)	−0.024 (0.0057)	0.30 (0.1747)	−0.007 (0.1533)	−0.33 (0.1586)	−0.005 (0.0084)
II	1.40 (0.0243)	−0.015 (0.0995)	0.60 (0.4523)	−0.011 (0.3354)	0.38 (0.3139)	−0.002 (0.6966)	−0.43 (0.1445)	0.003 (0.3433)
III	1.13 (0.0559)	−0.007 (0.4645)	1.00 (0.2039)	0.003 (0.8539)	0.33 (0.1424)	−0.007 (0.1488)	0.20 (0.4831)	0.003 (0.5821)
All the small-effect QTL (The 9th, 14th, 19th and 20th QTL)								
I	2.88 (0.0250)	−0.008 (0.2152)	2.75 (0.0792)	−0.008 (0.2152)	−0.25 (0.7027)	0.000 (1.000)	−0.38 (0.6084)	0.000 (1.0000)
II	3.38 (0.0265)	−0.013 (0.1942)	2.13 (0.0653)	−0.018 (0.1881)	1.00 (0.4228)	0.005 (0.4950)	−0.25 (0.7177)	0.000 (1.0000)
III	2.75 (0.1367)	−0.020 (0.0663)	2.75 (0.1946)	−0.015 (0.1817)	0.00 (1.0000)	−0.008 (0.0577)	0.00 (1.0000)	−0.003 (0.3910)
All the linked QTL (the 5th, 6th, 7th, 8th, 10th to 12th, and 16th to 18th QTL)								
I	1.65 (0.0128)	−0.040 (0.0262)	0.50 (0.5042)	−0.032 (0.0549)	0.70 (0.0607)	−0.014 (0.1216)	−0.45 (0.2620)	−0.006 (0.0811)
II	1.65 (0.1121)	−0.021 (0.2520)	0.35 (0.8222)	−0.007 (0.7505)	0.40 (0.5217)	−0.005 (0.4951)	−0.90 (0.0710)	−0.009 (0.1081)
III	0.60 (0.5231)	0.000 (1.0000)	0.60 (0.6717)	0.014 (0.6118)	0.65 (0.1027)	−0.01 (0.3107)	0.65 (0.1748)	0.004 (0.6618)

^*^All the probabilities in paired *t* test for differences of average powers or MADs across all the related QTL are in parentheses, where the difference equals to *A*−*B*.

**Table 2:** Main-effect DS3 QTL identified by genome-wide composite interval mapping (GCIM), composite interval mapping (CIM), empirical Bayes and joint multi-population analysis of Würschum *et al*. 27.

QTL	Chr	Posi (cM)	GCIM (new)				CIM					Empirical Bayes				Würschum *et al*.^27^		
			Marker interval	LOD	Additive	r^2^(%)	Marker interval	LOD	Additive	r^2^(%)	Population code	Marker	LOD	Additive	r^2^(%)	Marker	Additive	r^2^(%)
1	2A	61.16	wPt-6393~wPt-3114	14.32	−0.43	6.20	wPt-8826, wPt-3114~wPt-7466	2.63-7.25	−0.48-0.27	3.98~7.96	DH06,EAW74, EAW78	wPt-3114	12.76	−0.30	3.72	wPt-3114	−0.32	4.9
2	4A	14.7	wPt-6867	5.13	0.31	1.90												
3	4A	40.1	wPt-5428	3.19	−0.16	0.77	wPt-5857~wPt-5951	2.60	−0.54	20.16	DH07							
4	5A	2.5	wPt-5096	4.57	−0.29	1.39	wPt-5787~wPt-5096	2.77~5.08	−0.37~0.24	7.90~8.07	DH06,EAW74	wPt-5096	5.18	−0.33	2.26			
5	5A	47.85	wPt-7201~wPt-7769	4.07	−0.41	4.61										wPt-7255	−0.22	0.7
6	6A	14.5														wPt-4017	0.15	1.1
7	6A	38.8	wPt-3965	3.86	0.16	0.85												
8	6A	58.2	wPt-0902	7.96	−0.42	3.67	wPt-0902~tPt-513992	2.85~8.15	−0.52~1.27	8.49~13.85	DH06,EAW78	wPt-0902	7.48	−0.44	4.99	wPt-0902	−0.50	5.0
9	7A	12	tPt-512944	3.19	−0.16	0.83	rPt-389464~rPt-4199	2.75~5.02	−1.28~−0.37	7.91~13.84	DH06,EAW74	rPt-4199	3.49	−0.18	1.20			
10	7A	65.05	wPt-8377~wPt-7299	3.78	0.24	1.88						wPt-345934	3.99	0.21	1.43			
11	1B	38.02	wPt-0097~wPt-7476	2.85	−0.20	1.36	wPt-3765	2.83	0.22	6.92	DH06							
12	2B	130.9	wPt-6199~wPt-9958p2B	5.99	0.26	2.22						wPt-9958p2B	3.81	0.17	1.18	wPt-9958	0.20	1.9
13	3B	98.7					tPt-513153	2.41	0.37	6.52	EAW74					wPt-9422	−0.15	0.2
14	6B	50.73	wPt-5408~wPt-7426	3.51	−0.49	8.04										wPt-7426	−0.20	1.1
15	6B	76.5	wPt-3581	5.02	0.28	1.47	wPt-3581	6.56	0.40	17.16	DH07	wPt-3581	4.69	0.29	2.00	wPt-3581	0.30	1.4
16	7B	68.8	wPt-8919	4.84	−0.21	1.30	wPt-9798~wPt-9133	4.41	0.45	10.36	EAW74	wPt-9133	4.63	0.22	1.79			
17	3R	35.2					rPt-507396	2.55	−0.28	2.45	EAW78					rPt-507396	−0.33	0.9
18	4R	65.4	rPt-410866	5.00	−0.19	1.14	rPt-401323	2.68	−0.24	7.09	DH07	rPt-410866	3.70	−0.18	1.26	rPt-410866	−0.22	1.9
19	5R	18.9	rPt-399681	44.93	0.98	32.70	rPt-399681	27.27	1.02	35.02	EAW78	rPt-399681	38.78	0.98	40.27	rPt-399681	1.04	17.4
20	5R	35.2	rPt-402367	3.42	0.26	2.22										rPt-402367	0.30	1.8
21	6R	46.2	rPt-401125	5.97	−0.24	1.60	rPt-398551	2.61	1.28	13.86	DH06					rPt-401125	−0.24	1.4
22	7R	40.4					rPt-410852	2.77	−0.32	2.95	EAW78					rPt-400878	0.21	1.1

**Table 3:** Bayesian information criterion (BIC) for the regression of each trait on all the associated SNPs using genome-wide CIM (GCIM), empirical Bayes and joint analysis of Würschum *et al*. 27.

Trait	Twice the Negative logarithm likelihood function value			Bayesian information criterion (BIC)		
	GCIM	Empirical Bayes	Würschum *et al*.^27^	GCIM	Empirical Bayes	Würschum *et al*.^27^
DS1	2016.7	2192.2	2092.2	2210.9	2328.1	2292.8
DS2	2056.6	2152.9	2140.2	2179.6	2237.1	2250.3
DS3	1661.8	1764.5	1762.0	1797.7	1861.6	1872.0
