# Supplementary material for: Mapping small-effect and linked quantitative trait loci for complex traits in backcross or DH populations via a multi-locus GWAS methodology
Source: Sci Rep. 2016 Jul 20;6:29951. doi: 10.1038/srep29951 (PMC4951730; doi:10.1038/srep29951)
Supplement: Supplementary Information [file srep29951-s1.pdf]

Mapping small-effect and linked quantitative trait loci for complex traits in backcross or DH populations via a multi-locus GWAS methodology

Shi-Bo Wang[1,2], Yang-Jun Wen[2], Wen-Long Ren[2], Yuan-Li Ni[2], Jin Zhang[2], Jian-Ying Feng[2], Yuan-Ming Zhang[1]

[1]Statistical Genomics Lab, College of Plant Science and Technology, Huazhong Agricultural University, Wuhan 430070, China. [2]State Key Laboratory of Crop Genetics and Germplasm Enhancement, Nanjing Agricultural University, Nanjing 210095, China.

Correspondence and requests for materials should be addressed to Y.-M.Z. (email: soyzhang@mail.hzau.edu.cn)

Table S1 | True and estimated values for 20 simulated QTL parameters in backcross in simulation experiment I using GCIM under various QTL effect models and K matrices (200 replicates)

QTL	Position	Effect	r^2 (%)	Random QTL effect + K matrix from partial markers			Fixed QTL effect + K matrix from partial markers			Fixed QTL effect + K matrix from all the markers		
				Power (%)	Effect (MSE,MAD)	Position (MSE)	Power (%)	Effect (MSE,MAD)	Position (MSE)	Power (%)	Effect (MSE,MAD)	Position (MSE)
1	50	4.47	19.29	100	4.52(0.04,0.16)	50.14(1.05)	100	4.53(0.07,0.17)	50.14(1.06)	100	4.48(0.03,0.14)	49.95(0.35)
2	125	3.16	9.64	100	3.21(0.10,0.18)	125.05(1.19)	100	3.21(0.10,0.18)	125.03(1.19)	100	3.18(0.04,0.15)	125.12(0.72)
3	200	-2.24	4.85	99.5	-2.28(0.05,0.18)	200.05(1.51)	99.5	-2.27(0.06,0.19)	200.05(1.49)	100	-2.25(0.05,0.18)	200.01(1.03)
4	235	-1.58	2.41	98	-1.59(0.05,0.17)	234.85(3.28)	98	-1.59(0.05,0.17)	234.82(3.25)	98	-1.57(0.04,0.16)	234.98(2.94)
5	350	2.24	4.85	92.5	2.24(0.19,0.35)	349.65(1.90)	94	2.22(0.22,0.37)	349.56(1.94)	90	2.23(0.20,0.35)	350.69(2.89)
6	360	3.16	9.64	100	3.35(0.48,0.47)	360.05(1.51)	100	3.35(0.44,0.47)	360.09(1.59)	100	3.36(0.58,0.52)	359.37(1.76)
7	610	1.1	1.17	58.5	1.25(0.09,0.25)	609.57(4.55)	60.5	1.23(0.10,0.25)	609.60(4.68)	58.5	1.25(0.07,0.22)	609.91(3.87)
8	630	-1.1	1.17	60.5	-1.26(0.10,0.25)	630.45(3.96)	61.5	-1.25(0.11,0.26)	630.50(4.26)	62.5	-1.23(0.08,0.24)	630.36(3.43)
9	800	0.77	0.57	61.5	0.85(0.03,0.14)	800.19(5.05)	63	0.85(0.03,0.14)	800.16(5.05)	66	0.83(0.03,0.13)	800.26(5.12)
10	890	1.73	2.89	95	1.73(0.10,0.25)	889.99(2.69)	94	1.74(0.10,0.25)	890.03(2.74)	97.5	1.77(0.09,0.23)	890.40(2.96)
11	905	3.81	14.02	100	4.01(0.34,0.42)	905.03(1.05)	100	4.02(0.37,0.43)	905.08(1.10)	100	3.81(0.15,0.31)	905.06(0.89)
12	920	2.25	4.89	96	2.22(0.15,0.31)	920.02(1.86)	96.5	2.19(0.15,0.31)	920.06(1.86)	100	2.23(0.12,0.27)	919.66(1.92)
13	1100	-1.3	1.63	94.5	-1.34(0.03,0.14)	1099.87(2.67)	95	-1.34(0.03,0.15)	1099.86(2.68)	96.5	-1.33(0.03,0.14)	1099.97(2.51)
14	1210	-1	0.97	86.5	-1.02(0.03,0.14)	1210.05(4.47)	87	-1.02(0.03,0.14)	1210.08(4.69)	87.5	-1.02(0.03,0.14)	1210.17(4.51)
15	1305	-2.24	4.85	100	-2.28(0.06,0.20)	1304.86(1.67)	100	-2.28(0.07,0.21)	1304.84(1.70)	100	-2.25(0.05,0.18)	1304.89(1.28)
16	1335	1.58	2.41	88	2.21(0.59,0.68)	1335.70(2.89)	86.5	2.21(0.58,0.67)	1335.67(2.92)	88	2.18(0.54,0.65)	1335.69(2.83)
17	1345	1	0.97	26	1.88(1.03,0.88)	1344.33(3.13)	28	1.90(1.09,0.90)	1344.29(3.29)	28.5	1.81(0.97,0.81)	1344.40(3.12)
18	1365	-1.73	2.89	97	-1.62(0.10,0.26)	1365.30(2.29)	97	-1.63(0.10,0.27)	1365.33(2.21)	98	-1.62(0.10,0.26)	1365.20(1.87)
19	1800	0.71	0.49	59.5	0.81(0.03,0.14)	1799.97(5.31)	58	0.81(0.03,0.14)	1800.17(5.40)	63.5	0.79(0.03,0.12)	1799.93(7.36)
20	2300	0.89	0.76	77.5	0.94(0.03,0.13)	2300.19(3.36)	78.5	0.94(0.03,0.13)	2300.16(3.37)	80.5	0.93(0.03,0.13)	2300.22(4.13)

SD: standard deviation; MSE: mean squared error; MAD: mean absolute deviation. The same is true for the later tables.

Table S2 | True and estimated values for 20 simulated QTL parameters in backcross in simulation experiment II using GCIM under various QTL effect models and K matrices (200 replicates)

QTL	Position	Effect	r^2 (%)	Random QTL effect + K matrix from partial markers			Fixed QTL effect + K matrix from partial markers			Fixed QTL effect + K matrix from all the markers		
				Power (%)	Effect (MSE,MAD)	Position (MSE)	Power (%)	Effect (MSE,MAD)	Position (MSE)	Power (%)	Effect (MSE,MAD)	Position (MSE)
1	50	4.47	19.29	100	4.54(0.17,0.32)	50.12(1.02)	100	4.55(0.19,0.32)	50.07(1.02)	100	4.47(0.14,0.30)	50.03(0.45)
2	125	3.16	9.64	99.5	3.19(0.15,0.29)	125.02(1.33)	98	3.18(0.13,0.29)	125.02(1.32)	100	3.15(0.12,0.28)	124.99(0.84)
3	200	-2.24	4.85	99.5	-2.26(0.11,0.26)	200.21(1.64)	99	-2.25(0.13,0.28)	200.20(1.66)	99	-2.24(0.12,0.27)	200.08(1.51)
4	235	-1.58	2.41	96.5	-1.62(0.12,0.27)	234.89(2.72)	96.5	-1.63(0.12,0.28)	234.89(2.76)	97	-1.63(0.12,0.27)	234.99(2.52)
5	350	2.24	4.85	87	2.34(0.31,0.40)	349.51(1.86)	88	2.35(0.27,0.40)	349.52(1.84)	81	2.30(0.28.0.41)	350.49(2.78)
6	360	3.16	9.64	98.5	3.44(0.68,0.58)	359.90(1.64)	99.5	3.41(0.67,0.58)	360.03(1.56)	99	3.53(0.91,0.71)	359.18(2.05)
7	610	1.1	1.17	41.5	1.25(0.12,0.27)	609.54(3.16)	44.5	1.26(0.12,0.27)	609.58(2.69)	41.5	1.28(0.13,0.28)	609.64(3.01)
8	630	-1.1	1.17	39.5	-1.33(0.18,0.34)	630.37(4.16)	42.5	-1.31(0.15,0.30)	630.40(3.86)	42	-1.28(0.16,0.31)	630.06(3.54)
9	800	0.77	0.57	41	1.04(0.15,0.29)	799.72(4.99)	43	1.03(0.13,0.28)	799.93(5.26)	43.5	0.99(0.11,0.25)	800.21(4.78)
10	890	1.73	2.89	83	1.82(0.18,0.33)	890.20(2.81)	83.5	1.82(0.15,0.31)	890.20(2.93)	93.5	1.80(0.15,0.32)	890.56(3.20)
11	905	3.81	14.02	99.5	4.07(0.59,0.58)	904.97(1.13)	99.5	4.06(0.59,0.56)	904.96(1.16)	100	3.84(0.32,0.42)	905.06(1.01)
12	920	2.25	4.89	95	2.23(0.21,0.34)	919.83(2.33)	96	2.23(0.23,0.36)	919.85(2.43)	98	2.23(0.18,0.33)	919.42(2.49)
13	1100	-1.3	1.63	86	-1.34(0.10,0.25)	1099.98(3.74)	86.5	-1.34(0.10,0.25)	1100.03(3.77)	85	-1.33(0.09,0.23)	1099.82(3.48)
14	1210	-1	0.97	69	-1.15(0.11,0.26)	1209.84(4.10)	69	-1.14(0.11,0.26)	1209.81(4.09)	71.5	-1.16(0.12,0.26)	1209.85(4.24)
15	1305	-2.24	4.85	100	-2.28(0.13,0.28)	1304.98(1.72)	100	-2.28(0.12,0.28)	1304.95(1.73)	99	-2.26(0.12,0.28)	1304.99(1.44)
16	1335	1.58	2.41	78	2.29(0.72,0.75)	1335.59(2.65)	77	2.27(0.71,0.74)	1335.55(2.65)	81.5	2.27(0.68,0.73)	1335.52(2.91)
17	1345	1	0.97	29	2.10(1.44,1.10)	1344.22(2.95)	30.5	2.09(1.42,1.09)	1344.18(2.95)	26	2.03(1.34,1.03)	1344.27(2.73)
18	1365	-1.73	2.89	92.5	-1.67(0.14,0.30)	1365.32(1.93)	91.5	-1.68(0.14,0.29)	1365.33(1.95)	93.5	-1.65(0.14,0.29)	1365.19(1.59)
19	1800	0.71	0.49	40.5	1.05(0.20,0.35)	1799.67(5.15)	39.5	1.05(0.19,0.35)	1799.79(4.90)	43.5	1.02(0.17,0.31)	1799.53(5.09)
20	2300	0.89	0.76	54.5	1.06(0.08,0.22)	2299.74(4.51)	54.5	1.07(0.09,0.23)	2299.77(4.67)	56	1.05(0.09,0.23)	2299.76(4.62)

Table S3 | True and estimated values for 20 simulated QTL parameters in backcross in simulation experiment III using GCIM under various QTL effect models and K matrices (200 replicates)

QTL	Position	Effect	r^2 (%)	Random QTL effect + K matrix from partial markers			Fixed QTL effect + K matrix from partial markers			Fixed QTL effect + K matrix from all the markers		
				Power (%)	Effect (MSE,MAD)	Position (MSE)	Power (%)	Effect (MSE,MAD)	Position (MSE)	Power (%)	Effect (MSE,MAD)	Position (MSE)
1	50	4.47	19.29	100	4.53(0.12,0.25)	50.10(1.14)	100	4.52(0.12,0.25)	50.09(1.15)	100	4.50(0.11,0.24)	50.04(0.61)
2	125	3.16	9.64	99	3.20(0.08,0.23)	124.95(1.67)	99.5	3.21(0.09,0.23)	125.00(1.66)	100	3.18(0.09,0.23)	125.01(1.31)
3	200	-2.24	4.85	99	-2.34(0.16,0.31)	200.11(2.76)	99	-2.33(0.15,0.29)	200.09(2.76)	98.5	-2.30(0.13,0.29)	199.97(2.45)
4	235	-1.58	2.41	84	-1.61(0.10,0.26)	234.68(4.02)	85.5	-1.62(0.10,0.26)	234.67(4.05)	86	-1.61(0.11,0.27)	234.75(3.87)
5	350	2.24	4.85	70.5	2.53(0.47,0.48)	349.87(2.34)	72	2.57(0.63,0.52)	349.84(2.41)	62	2.68(0.86,0.59)	350.85(3.48)
6	360	3.16	9.64	98	3.63(1.36,0.88)	359.96(1.95)	96.5	3.63(1.33,0.86)	360.01(1.95)	95	3.80(1.72,1.03)	359.43(2.22)
7	610	1.1	1.17	12	1.54(0.31,0.46)	608.88(5.13)	12	1.51(0.29,0.43)	608.88(5.04)	15.5	1.45(0.22,0.39)	609.19(5.00)
8	630	-1.1	1.17	11	-1.50(0.25,0.44)	630.36(4.55)	11.5	-1.47(0.23,0.42)	630.13(3.52)	12	-1.56(0.32,0.52)	630.13(2.71)
9	800	0.77	0.57	25.5	1.09(0.12,0.32)	800.31(3.57)	27	1.09(0.12,0.32)	800.26(3.44)	27.5	1.07(0.11,0.30)	800.36(4.55)
10	890	1.73	2.89	69	1.95(0.18,0.33)	890.49(3.91)	66.5	1.95(0.16,0.33)	890.55(4.25)	70.5	1.96(0.17,0.32)	890.92(4.30)
11	905	3.81	14.02	99.5	4.27(0.19,0.83)	904.83(1.60)	100	4.32(1.33,0.88)	904.85(1.56)	99.5	4.12(1.00,0.74)	904.96(1.43)
12	920	2.25	4.89	93	2.28(0.24,0.38)	919.79(3.26)	91	2.29(0.20,0.36)	919.77(3.28)	95	2.28(0.18,0.34)	919.25(3.55)
13	1100	-1.3	1.63	79	-1.38(0.08,0.22)	1100.15(4.10)	80	-1.38(0.08,0.22)	1100.14(4.04)	81.5	-1.36(0.07,0.21)	1100.13(4.40)
14	1210	-1	0.97	46.5	-1.17(0.06,0.19)	1210.22(5.20)	46	-1.17(0.06,0.19)	1210.24(5.28)	52.5	-1.17(0.07,0.20)	1210.13(6.50)
15	1305	-2.24	4.85	97	-2.28(0.20,0.35)	1305.03(2.72)	96.5	-2.27(0.20,0.35)	1304.98(2.71)	97.5	-2.26(0.18,0.33)	1305.10(2.61)
16	1335	1.58	2.41	75	2.29(0.73,0.75)	1335.63(3.45)	73.5	2.29(0.71,0.74)	1335.67(3.44)	77	2.27(0.68,0.73)	1335.63(3.63)
17	1345	1	0.97	19	2.33(2.02,1.33)	1343.84(5.26)	19.5	2.30(1.98,1.30)	1343.97(4.77)	18.5	2.31(1.97,1.31)	1344.38(4.73)
18	1365	-1.73	2.89	78	-1.65(0.12,0.28)	1365.74(3.27)	76	-1.66(0.11,0.28)	1365.72(3.16)	79.5	-1.63(0.12,0.29)	1365.55(3.14)
19	1800	0.71	0.49	22.5	1.05(0.14,0.34)	1799.47(7.64)	23	1.05(0.14,0.34)	1799.35(6.96)	22.5	1.03(0.12,0.32)	1799.33(7.64)
20	2300	0.89	0.76	34	1.16(0.11,0.27)	2300.12(5.06)	32.5	1.16(0.11,0.28)	2300.02(4.60)	37	1.13(0.10,0.25)	230.10(5.50)

Table S4 | Comparison of genome-wide composite interval mapping (CIM) with CIM and empirical Bayes in backcross with 400 individuals and 20 simulated main-effect QTL (200 replicates)

QTL	Position	Effect	r^2 (%)	Genome-wide CIM (GCIM)			CIM			Empirical Bayes		
				Power(%)	Effect(SD,MSE,MAD)	Position(SD,MSE)	Power(%)	Effect(SD,MSE,MAD)	Position(SD,MSE)	Power(%)	Effect(SD,MSE,MAD)	Position(SD,MSE)
1	50	4.47	19.29	100	4.47(0.18,0.03,0.14)	49.99(0.58,0.34)	99.5	4.50(0.28,0.08,0.22)	49.95(0.54,0.30)	100	4.47(0.17,0.03,0.14)	50(0,0)
2	125	3.16	9.64	100	3.17(0.19,0.04,0.15)	125.1(0.84,0.71)	99.5	3.05(0.37,0.15,0.31)	124.97(0.97,0.94)	100	3.14(0.19,0.04,0.15)	124.99(0.20,0.04)
3	200	-2.24	4.85	100	-2.25(0.23,0.05,0.18)	200.01(1.01,1.03)	94.5	-2.81(0.57,0.64,0.70)	200.62(1.15,1.69)	100	-2.24(0.20,0.04,0.16)	200(0,0)
4	235	-1.58	2.41	98	-1.58(0.20,0.04,0.16)	234.97(1.73,2.98)	84	-1.78(0.63,0.44,0.45)	234.78(1.86,3.51)	99	-1.56(0.19,0.04,0.15)	234.97(1.55,2.40)
5	350	2.24	4.85	91.5	2.23(0.42,0.17,0.33)	350.53(1.54,2.63)	73.5	4.88(0.31,7.04,2.64)	352.55(1.10,7.71)	99.5	2.23(0.29,0.08,0.24)	350.02(0.35,0.13)
6	360	3.16	9.64	100	3.34(0.68,0.50,0.47)	359.45(1.15,1.62)	94	4.98(0.29,3.41,1.82)	358.46(1.02,3.41)	100	3.16(0.30,0.09,0.23)	360.01(0.13,0.02)
7	610	1.1	1.17	60.5	1.25(0.24,0.08,0.23)	609.88(2.06,4.21)	1	0.98(0.03,0.01,0.12)	607(1.41,10)	54	1.22(0.21,0.06,0.20)	610.05(1.99,3.94)
8	630	-1.1	1.17	64	-1.24(0.26,0.09,0.24)	630.29(1.96,3.90)	2	-0.98(0.04,0.02,0.12)	632.5(1.91,9)	56.5	-1.22(0.23,0.07,0.22)	630.04(1.95,3.76)
9	800	0.77	0.57	66	0.83(0.15,0.03,0.13)	800.17(2.33,5.43)	35	1.15(0.16,0.17,0.38)	800.74(2.79,8.23)	64	0.83(0.14,0.02,0.12)	800.20(2.46,6.05)
10	890	1.73	2.89	98.5	1.78(0.29,0.08,0.23)	890.34(1.68,2.93)	77	2.04(0.46,0.30,0.39)	889.72(1.39,1.99)	100	1.75(0.25,0.06,0.20)	889.88(0.93,0.88)
11	905	3.81	14.02	100	3.79(0.40,0.16,0.31)	905.08(0.88,0.78)	98.5	6.83(0.45,9.30,3.03)	905.11(1.06,1.32)	100	3.82(0.32,0.10,0.25)	905(0,0)
12	920	2.25	4.89	100	2.23(0.33,0.11,0.26)	919.69(1.33,1.86)	91.5	2.41(0.94,0.91,0.49)	919.82(1.39,1.96)	100	2.22(0.27,0.07,0.22)	920(0,0)
13	1100	-1.3	1.63	96.5	-1.33(0.17,0.03,0.14)	1100.04(1.57,2.46)	87	-1.46(0.24,0.08,0.23)	1100.13(1.97,3.87)	96.5	-1.32(0.16,0.03,0.13)	1099.97(1.20,1.42)
14	1210	-1	0.97	88	-1.02(0.17,0.03,0.14)	1210.08(2.19,4.78)	74.5	-1.44(0.26,0.26,0.44)	1209.92(2.39,5.69)	89	-1..01(0.17,0.03,0.13)	1209.94(2.38,5.62)
15	1305	-2.24	4.85	100	-2.25(0.24,0.06,0.19)	1304.88(1.13,1.29)	94	-1.65(0.25,0.41,0.59)	1304.11(1.24,2.32)	100	-2.22(0.25,0.06,0.20)	1304.98(0.35,0.13)
16	1335	1.58	2.41	90	2.17(0.43,0.53,0.65)	1335.68(1.56,2.88)	0			96	1.95(0.44,0.33,0.49)	1335.40(1.70,3.02)
17	1345	1	0.97	27.5	1.74(0.55,0.84,0.74)	1344.49(1.62,2.84)	0.05	0.90(0,0.01,0.10)	1340(0,25)	35	1.39(0.32,0.26,0.40)	1344.97(0.29,0.08)
18	1365	-1.73	2.89	98	-1.61(0.29,0.10,0.26)	1365.23(1.37,1.93)	50	-1.14(0.18,0.38,0.60)	1365.83(1.69,3.51)	98.5	-1.56(0.32,0.13,0.29)	1365.03(0.94,0.89)
19	1800	0.71	0.49	61.5	0.79(0.14,0.03,0.12)	1799.86(2.74,7.44)	16.5	1.07(0.11,0.14,0.36)	1799.61(2.66,7)	61.5	0.79(0.13,0.02,0.11)	1800.08(3.00,8.94)
20	2300	0.89	0.76	81	0.93(0.16,0.03,0.13)	2300.31(2.05,4.29)	45	1.13(0.16,0.08,0.24)	2299.81(2.25,5.06)	83.5	0.93(0.15,0.02,0.12)	2300.51(2.24,5.24)

Table S5 | Comparison of genome-wide composite interval mapping (CIM) with CIM and empirical Bayes in backcross with 400 individuals, 20 simulated QTL and polygenic background (200 replicates)

QTL	Position	Effect	r^2 (%)	Genome-wide CIM (GCIM)			CIM			Empirical Bayes		
				Power(%)	Effect(SD,MSE,MAD)	Position(SD,MSE)	Power(%)	Effect(SD,MSE,MAD)	Position(SD,MSE)	Power(%)	Effect(SD,MSE,MAD)	Position(SD,MSE)
1	50	4.47	18.41	100	4.47(0.37,0.14,0.30)	50.04(0.66,0.44)	99.5	4.49(0.40,0.16,0.32)	50.03(0.75,0.56)	100	4.44(0.38,0.14,0.30)	50.01(0.10,0.01)
2	125	3.16	9.20	100	3.14(0.36,0.13,0.28)	124.96(0.90,0.81)	100	2.98(0.48,0.26,0.42)	124.88(1.07,1.15)	100	3.12(0.34,0.11,0.27)	125(0,0)
3	200	-2.24	4.62	99.5	-2.23(0.34,0.12,0.27)	200.09(1.22,1.49)	89	-2.74(0.61,0.62,0.66)	200.55(1.58,2.79)	99	-2.20(0.33,0.11,0.26)	200.05(0.71,0.51)
4	235	-1.58	2.30	96.5	-1.63(0.34,0.12,0.27)	235.05(1.59,2.52)	79.5	-1.95(0.66,0.57,0.53)	234.62(1.70,3.01)	97	-1.63(0.32,0.10,0.25)	234.90(1.13,1.29)
5	350	2.24	4.62	86	2.27(0.49,0.24,0.39)	350.34(1.58,2.60)	71.5	4.97(0.62,7.84,2.73)	352.39(1.22,7.18)	98	2.28(0.40,0.16,0.32)	349.92(0.94,0.89)
6	360	3.16	9.20	99.5	3.45(0.83,0.76,0.65)	359.32(1.14,1.74)	91	5.03(0.38,3.65,1.87)	358.45(1.11,3.62)	100	3.19(0.48,0.23,0.35)	359.99(0.16,0.03)
7	610	1.1	1.11	42	1.23(0.32,0.12,0.27)	609.62(1.70,3.00)	6.5	1.17(0.20,0.04,0.14)	609.62(0.51,0.38)	38.5	1.21(0.28,0.09,0.24)	609.48(1.54,2.60)
8	630	-1.1	1.11	41	-1.29(0.35,0.16,0.31)	629.88(1.79,3.17)	5	-1.16(0.23,0.05,0.16)	631.4(1.78,4.8)	39	-1.21(0.31,0.11,0.27)	630.67(3.75,14.32)
9	800	0.77	0.55	44.5	1.00(0.27,0.12,0.26)	800.03(2.38,5.58)	29.5	1.29(0.26,0.33,0.52)	799.95(2.69,7.10)	43	1.02(0.27,0.13,0.28)	800.12(2.76,7.56)
10	890	1.73	2.76	91	1.81(0.39,0.16,0.33)	890.44(1.67,2.96)	67.5	2.28(0.84,1.00,0.62)	889.73(1.63,2.70)	97	1.78(0.34,0.12,0.28)	889.95(1.35,1.80)
11	905	3.81	13.37	100	3.87(0.59,0.34,0.44)	905.09(0.98,0.96)	99.5	6.79(0.69,9.33,3.00)	905.08(1.19,1.41)	100	3.81(0.44,0.20,0.33)	905.83(0.14,0.02)
12	920	2.25	4.66	98.5	2.22(0.41,0.17,0.33)	919.57(1.43,2.21)	86	2.51(0.90,0.87,0.54)	919.84(1.48,2.19)	99.5	2.20(0.38,0.14,0.30)	920(0.71,0.50)
13	1100	-1.3	1.56	84.5	-1.33(0.31,0.10,0.24)	1099.88(1.90,3.59)	72	-1.58(0.42,0.25,0.36)	1100.26(1.94,3.79)	79.5	-1.30(0.30,0.09,0.24)	1099.94(2.05,4.19)
14	1210	-1	0.92	73.5	-1.16(0.30,0.11,0.26)	1209.88(2.09,4.34)	63.5	-1.55(0.36,0.43,0.55)	1210.03(2.54,6.38)	71	-1.14(0.29,0.10,0.25)	1209.93(2.15,4.58)
15	1305	-2.24	4.62	99	-2.26(0.34,0.12,0.27)	1304.97(1.19,1.41)	90.5	-1.68(0.36,0.45,0.58)	1304.09(1.38,2.71)	99.5	-2.20(0.35,0.12,0.28)	1305.05(0.71,0.50)
16	1335	1.58	2.30	81.5	2.27(0.45,0.68,0.73)	1335.49(1.65,2.93)	2	1.27(0.25,0.14,0.31)	1336.25(2.99,8.25)	88.5	2.06(0.46,0.44,0.60)	1335.31(1.78,3.25)
17	1345	1	0.92	26	2.03(0.52,1.32,1.03)	1344.29(1.51,2.75)	1	1.07(0.17,0.02,0.12)	1342.5(3.54,12.5)	27	1.65(0.42,0.59,0.68)	1345.09(0.68,0.46)
18	1365	-1.73	2.76	94.5	-1.64(0.38,0.15,0.30)	1365.21(1.25,1.60)	40.5	-1.29(0.22,0.24,0.44)	1365.99(1.76,4.05)	94	-1.56(0.35,0.15,0.31)	1365.16(1.02,1.06)
19	1800	0.71	0.46	41.5	1.03(0.27,0.17,0.33)	1799.49(2.36,5.76)	18	1.31(0.23,0.41,0.59)	1800.08(2.62,6.69)	44	0.99(0.26,0.15,0.28)	1799.55(2.80,7.95)
20	2300	0.89	0.73	59	1.05(0.24,0.08,0.22)	2299.84(2.23,4.97)	33.5	1.27(0.22,0.20,0.38)	2299.90(2.12,4.43)	55	1.05(0.24,0.08,0.22)	2299.64(2.32,5.45)

Table S6 | Comparison of genome-wide composite interval mapping (CIM) with CIM and empirical Bayes in backcross with 400 individuals, 20 QTL and 3 interactions (200 replicates)

QTL	Position	Effect	r^2 (%)	Genome-wide CIM (GCIM)			CIM			Empirical Bayes		
				Power(%)	Effect(SD,MSE,MAD)	Position(SD,MSE)	Power(%)	Effect(SD,MSE,MAD)	Position(SD,MSE)	Power(%)	Effect(SD,MSE,MAD)	Position(SD,MSE)
1	50	4.47	17.27	100	4.50(0.32,0.10,0.24)	50.04(0.78,0.61)	100	4.53(0.34,0.12,0.28)	49.97(0.74,0.55)	100	4.47(0.30,0.09,0.23)	50(0,0)
2	125	3.16	8.63	100	3.19(0.29,0.08,0.23)	125.03(1.14,1.29)	99.5	2.99(0.45,0.23,0.40)	124.93(1.11,1.24)	100	3.14(0.28,0.08,0.22)	125(0.50,0.25)
3	200	-2.24	4.34	98.5	-2.31(0.37,0.14,0.29)	199.97(1.56,2.42)	90	-2.78(0.55,0.59,0.66)	200.58(1.61,2.9)	99	-2.26(0.35,0.12,0.27)	200.10(1.33,1.77)
4	235	-1.58	2.16	86	-1.61(0.32,0.10,0.26)	234.74(2.02,4.12)	60	-2.13(0.80,0.93,0.66)	234.46(1.90,3.88)	87	-1.61(0.31,0.10,0.25)	234.74(2.17,4.74)
5	350	2.24	4.34	65	2.62(0.76,0.72,0.56)	350.7(1.65,3.21)	71.5	4.92(0.33,7.27,2.68)	352.30(1.20,6.73)	91.5	2.31(0.40,0.17,0.34)	350.05(0.91,0.82)
6	360	3.16	8.63	95.5	3.72(1.11,1.55,0.96)	359.53(1.32,1.95)	91.5	5.02(0.33,3.56,1.86)	358.32(1.11,4.04)	99.5	3.20(0.65,0.42,0.46)	359.95(0.50,0.25)
7	610	1.1	1.05	15	1.47(0.33,0.24,0.41)	609.13(2.15,5.2)	1.5	1.07(0.04,0.002,0.04)	610(0,0)	8.5	1.40(0.30,0.18,0.33)	608.53(2.35,7.35)
8	630	-1.1	1.05	13	-1.52(0.34,0.29,0.48)	630.04(1.91,3.5)	1.5	-1.19(0.003,0.01,0.09)	630(0,0)	6.5	-1.52(0.31,0.27,0.45)	629.62(1.39,1.92)
9	800	0.77	0.51	29	1.06(0.15,0.11,0.29)	800.34(2.32,5.41)	21	1.36(0.31,0.43,0.59)	800.11(2.51,6.15)	24.5	1.05(0.13,0.10,0.28)	800(2.70,7.14)
10	890	1.73	2.59	71	1.97(0.33,0.16,0.32)	890.84(1.85,4.11)	53.5	2.18(0.40,0.36,0.47)	889.62(1.41,2.10)	83	1.86(0.32,0.12,0.28)	890.15(1.60,2.56)
11	905	3.81	12.55	100	4.10(0.90,0.90,0.72)	904.98(1.19,1.41)	99.5	6.86(0.48,9.53,3.08)	905.08(1.24,1.53)	99.5	3.97(0.69,0.50,0.53)	904.98(0.42,0.17)
12	920	2.25	4.38	97	2.26(0.42,0.18,0.34)	919.38(1.73,3.36)	82.5	2.47(0.83,0.74,0.49)	920.02(1.59,2.52)	99	2.24(0.50,0.25,0.36)	919.95(1.42,2.02)
13	1100	-1.3	1.46	81.5	-1.36(0.25,0.07,0.21)	1100.28(2.13,4.57)	71	-1.52(0.28,0.12,0.27)	1100.04(2.06,4.20)	82.5	-1.37(0.26,0.07,0.22)	1100.09(2.37,5.61)
14	1210	-1	0.86	52	-1.17(0.19,0.06,0.19)	1210.15(2.64,6.94)	63.5	-1.54(0.32,0.40,0.54)	1209.96(2.47,6.04)	52	-1.17(0.19,0.06,0.20)	1210.19(3.19,10.10)
15	1305	-2.24	4.34	97.5	-2.55(0.43,0.18,0.33)	1305.10(1.61,2.61)	89	-1.65(0.31,0.44,0.60)	1304.33(1.33,2.20)	98	-2.18(0.42,0.18,0.33)	1305.03(1.56,2.42)
16	1335	1.58	2.16	76.5	2.28(0.44,0.68,0.74)	1335.62(1.81,3.65)	0			80	2.10(0.45,0.47,0.59)	1335.69(2.21,5.31)
17	1345	1	0.86	18	2.33(0.49,2.00,1.33)	1344.56(1.99,4.06)	0			13	2.23(0.51,1.75,1.23)	1345.58(1.63,2.88)
18	1365	-1.73	2.59	80	-1.62(0.34,0.12,0.30)	1365.52(1.70,3.14)	24.5	-1.31(0.23,0.23,0.46)	1365.69(1.75,3.47)	72	-1.57(0.34,0.14,0.31)	1365.37(1.85,3.55)
19	1800	0.71	0.44	21.5	1.02(0.14,0.12,0.31)	1799.47(3.06,9.42)	12	1.29(0.32,0.43,0.58)	1800.17(3.23,10.00)	20	1.06(0.15,0.15,0.35)	1799.63(2.86,8.13)
20	2300	0.89	0.68	37	1.14(0.20,0.10,0.25)	2300.19(2.54,6.38)	21	1.35(0.40,0.37,0.46)	2300.18(2.85,7.93)	33.5	1.13(0.17,0.09,0.24)	2300.60(2.82,8.21)

Table S7 | Main-effect QTL identified by GCIM and some common QTL identified by CIM, ICIM and empirical Bayes.

Trait	Chr	Posi (cM)	Genome-wide CIM (GCIM)				CIM					Empirical Bayes (multi-marker analysis)				Würschum *et al.* (2014)		
			Marker interval	LOD	Additive	r^2(%)	Marker interval	LOD	Additive	r^2(%)	Population	marker	LOD	Additive	r^2(%)	Marker	Effect	r^2(%)
DS1	1A	79.8	wPt-7339	3.49	0.23	0.67						wPt-7339	4.81	0.30	1.39			
	2A	48.9	wPt-7026													wPt-7026	-0.23	0.2
	2A	55.8	rPt-505274~wPt5251	8.93	-0.42	2.53	wPt-6393~wPt3114, wPt-5251	2.62~4.24	-0.93~0.42	5.01~28.78	DH07,EAW74							
	2A	62.1					wPt-3114~wPt-7466	4.29	-0.61	10.38	DH06	wPt-3114	7.66	-0.32	1.68	wPt-3114	-0.30	2.2
	4A	17.1	tPt-512917	4.87	0.40	1.42						tPt-512917	4.98	0.44	1.99	tPt-512917	0.43	1.7
	4A	41.1	wPt-5857	3.79	-0.26	0.87	wPt-5857~wPt-5951	2.66~3.30	-0.89~-0.65	12.93~13.41	DH07,EAW74					wPt-5857	-0.31	0.6
	4A	103.8														wPt-7391	-0.19	0.7
	5A	34.5	tPt-3642	3.91	0.32	0.90						wPt-2329	2.98	0.34	1.20	wPt-2329	0.29	0.9
	6A	4.7	wPt-8443~wPt-9832	4.37	-0.93	12.00												
	6A	14.7														wPt-7330	0.27	1.0
	6A	62.4	tPt-4209	17.60	0.56	3.55	wPt0902,tPt-4209	2.66~10.97	-0.75~0.33	3.11~10.89	EAW74,EAW78	tPt-4209	13.80	0.55	4.01	tPt-4209	0.46	4.1
	7A	53.2					wPt-7785	2.92	-0.35	2.35	EAW78					wPt-7785	-0.18	0.6
	7A	65.1	wPt-8377~wPt-7299	3.53	0.30	1.27	wPt-1961	4.04	0.41	3.21	EAW78							
	7A	118.9	wPt-4489~wPt0494p7A	2.84	0.22	0.66										wPt-0494	0.21	0.9
	1B	26.2	wPt-2526~wPt4532p1B	5.22	0.34	1.60						wPt-2526	3.72	0.26	1.04	wPt-5003	0.45	1.8

2B	148.6	tPt-1663	7.50	0.35	1.42	tPt-1663,wPt-0100~ wPt-9274	2.77~4.85	0.44~0.54	5.61~6.00	EAW74,EAW7 8	tPt-1663	6.69	0.39	2.01	tPt-1663	0.39	2.9
3B	98.7	wPt-9422	5.11	-0.28	0.97										tPt-513153	0.15	0.4
4B	54.9					wPt-6016	3.51	0.37	3.87	EAW74					wPt-6016	0.33	0.7
5B	39.9	wPt-1548	11.46	0.63	3.02	wPt-4936	7.42	-0.59	7.08	EAW78	wPt-7101	6.27	-0.42	2.70	wPt-1548	0.52	1.5
6B	5	wPt-3304	3.90	0.28	0.89										wPt-3304	0.32	0.6
6B	53.2					wPt-8721	3.67	-0.54	7.12	DH06					wPt-2400	0.20	0.7
6B	62.7	wPt-5037~wPt9124	7.17	-0.38	1.80	wPt-8554~tPt-3689	3.46~7.95	0.53~0.79	7.28~20.63	DH06,DH07							
6B	76.5					wPt-3581	9.38	0.89	22.34	DH07					wPt-3581	0.33	1.5
7B	68.8	wPt-8919	7.09	-0.35	1.44	wPt-8919	5.70	-0.49	6.76	EAW74	wPt-8919	6.72	-0.38	2.00	wPt-1149	0.52	2.8
4R	9.9	rPt-389770	7.89	0.65	5.00	wPt-8336	10.12	0.90	21.64	DH06	rPt-389770	5.25	0.63	5.56	rPt-389770	0.60	1.5
4R	44	rPt-507237	11.60	0.39	2.11	tPt-513924, rPt-507237	4.49~6.07	-0.50~0.46	4.06~7.16	EAW74,EAW7 8	rPt-507237	7.25	0.36	2.11	rPt-509552	0.48	2.0
4R	66.3	rPt-390324	17.23	-0.74	4.82	tPt-513520,rPt-2245	4.91~6.35	-0.58~0.70	11.44~12.72	DH06,DH07	rPt-390324	17.01	-0.83	7.21	rPt-506436	0.51	2.3
5R	18.9	rPt-399681	41.49	1.27	22.82	rPt-399681	33.18	1.42	41.87	EAW78	rPt-399681	37.50	1.32	29.23	rPt-399681	1.33	13.0
5R	36.5	rPt-508041	4.93	0.40	2.32	rPt-508041~rPt-398691	4.09	0.64	9.83	EAW74					rPt-508041	0.38	0.7
5R	62.9	rPt-507480	4.44	-0.30	1.28												
5R	81.3														rPt-505265	0.09	0.1
6R	39.6	tPt-4479	8.54	0.43	1.89	rPt-8205	14.35	0.84	19.63	EAW74	tPt-4479	4.26	0.51	3.14			
6R	41.2	tPt-507562	11.75	-0.40	2.16	rPt-390525	2.74	0.35	2.48	EAW78					rPt-401125	-0.54	3.5

	6R	62.8					rPt-399543	2.98	0.36	2.75	EAW78					rPt-508379	0.24	0.5
	6R	72.7	rPt-390698	5.69	-0.39	1.30										rPt-390698	0.43	0.9
	7R	40.7	rPt-401882	4.86	0.32	1.18												
	7R	43.3	rPt-390741	4.24	-0.31	1.25						rPt-390741	3.41	-0.31	1.47	rPt-390741	-0.35	1.4
DS2	2A	62.1	wPt-3114	5.41	-0.24	0.93	wPt-6393	5.90	0.43	8.00	EAW74	wPt-3114	5.16	-0.26	1.49	wPt-3114	0.30	2.9
	6A	15.6	wPt-9075	3.98	-0.25	0.90						wPt-9075	3.82	-0.25	1.26			
	6A	58.2	wPt-0902	9.19	-0.59	3.43	tPt-513137~tPt-513992	3.85~6.48	-2.78~1.42	13.19~19.88	DH06,DH07,EAW78,	tPt-4209	8.05	0.41	3.04	wPt-2077	0.69	2.7
	7A	66	wPt-7299	4.25	0.31	1.25	wPt-8337	6.53	1.52	26.35	DH06					wPt-7299	0.35	1.5
	2B	148.6	tPt-1663	9.72	0.41	2.22	tPt-512890~wPt2106, wPt-2106,tPt-1663	2.74~5.13	-1.66~0.61	3.56~21.52	DH06,EAW74, EAW78	tPt-1663	10.89	0.46	3.87			
	2B	151.05	wPt-0100~wPt-9274	2.99	1.25	25.37	rPt-398598	5.98	0.66	7.02	EAW78							
	3B	0	wPt-9496	2.57	-0.23	0.60	wPt-9496~wPt-2426p3B	3.92	1.47	16.01	EAW74	wPt-9496	3.55	-0.30	1.43			
	3B	98.7	wPt-9422	3.33	-0.23	0.74										wPt-9422	-0.27	0.4
	5B	39.9	wPt-1548	6.64	0.49	2.06	wPt-4936,wPt-2607~ wPt-1548,wPt-7101	3.85~7.71	-1.48~1.42	4.89~27.52	DH06,EAW74, EAW78	wPt-1548	5.80	0.47	2.71	wPt-1548	0.43	1.6
	5B	59.1	wPt-1733	4.22	0.38	1.32	wPt-9300,wPt-6265	4.25~5.79	-1.65~0.35	5.62~21.78	DH06,EAW74					wPt-1733	0.45	1.3
	6B	76.5	wPt-3581	4.81	0.37	1.23	tPt-514287~tPt-9132, wPt-3581	7.35~8.99	-1.19~0.57	20.94~33.63	DH06,DH07					wPt-3581	0.33	1.3
	6B	116.95	wPt-0406~rPt-505542	3.30	0.49	3.81	tPt-513425	4.36	1.49	17.69	DH06					rPt-505542	0.22	1.1
	7B	67.4	wPt-8312	4.42	0.28	1.05	wPt-9133,wPt-8312, wPt-1149	3.03~5.61	-1.54~2.14	6.99~20.48	DH06,EAW74, EAW78	wPt-8312	3.92	0.28	1.49			

	3R	35.4														rPt-402572	-0.22	0.8
	4R	63.9	rPt-505489	10.26	0.42	2.42	tPt-4576,rPt-398587	2.85~5.81	-1.31~2.48	14.47~15.30	DH07,EAW74, EAW78					rPt-509321,rPt-389618	-0.50~-0.49	0.1~3.1
	5R	18.9	rPt-399681	44.26	1.33	28.40	rPt-507500	22.73	1.52	37.9	EAW78	rPt-399681	41.15	1.37	42.92	rPt-399681	1.45	18.3
	6R	41.2					rPt-507115	8.47	-0.53	12.52	EAW74					rPt-507562	-0.36	2.6
	7R	46.9	wPt-1598~rPt-505136	7.47	0.31	1.51	rPt-400849	4.42	1.55	17.96	DH07					rPt-401147	0.39	0.8
DS3	2A	61.16	wPt-6393~wPt-3114	14.32	-0.43	6.20	wPt-8826,wPt-3114~ wPt-7466	2.63-7.25	-0.48-0.27	3.98~7.96	DH06,EAW74, EAW78	wPt-3114	12.76	-0.30	3.72	wPt-3114	-0.32	4.9
	4A	14.7	wPt-6867	5.13	0.31	1.90												
	4A	40.1	wPt-5428	3.19	-0.16	0.77	wPt-5857~wPt-5951	2.60	-0.54	20.16	DH07							
	5A	2.5	wPt-5096	4.57	-0.29	1.39	wPt-5787,wPt-5096	2.77~5.08	-0.37~0.24	7.90~8.07	DH06,EAW74	wPt-5096	5.18	-0.33	2.26			
	5A	47.85	wPt-7201~wPt-7769	4.07	-0.41	4.61										wPt-7255	-0.22	0.7
	6A	14.5														wPt-4017	0.15	1.1
	6A	38.8	wPt-3965	3.86	0.16	0.85												
	6A	58.2	wPt-0902	7.96	-0.42	3.67	wPt-0902,tPt-513992	2.85~8.15	-0.52~1.27	8.49~13.85	DH06,EAW78	wPt-0902	7.48	-0.44	4.99	wPt-0902	-0.50	5.0
	7A	12	tPt-512944	3.19	-0.16	0.83	rPt-389464,rPt-4199	2.75~5.02	-1.28~-0.37	7.91~13.84	DH06,EAW74	rPt-4199	3.49	-0.18	1.20			
	7A	65.05	wPt-8377~wPt-7299	3.78	0.24	1.88						wPt-345934	3.99	0.21	1.43			
	1B	38.02	wPt-0097~wPt-7476	2.85	-0.20	1.36	wPt-3765	2.83	0.22	6.92	DH06							
	2B	130.9	wPt-6199~wPt-9958p2B	5.99	0.26	2.22						wPt-9958p2B	3.81	0.17	1.18	wPt-9958	0.20	1.9
	3B	98.7					tPt-513153	2.41	0.37	6.52	EAW74					wPt-9422	-0.15	0.2

6B	50.73	wPt-5408~wPt-7426	3.51	-0.49	8.04										wPt-7426	-0.20	1.1
6B	76.5	wPt-3581	5.02	0.28	1.47	wPt-3581	6.56	0.40	17.16	DH07	wPt-3581	4.69	0.29	2.00	wPt-3581	0.30	1.4
7B	68.8	wPt-8919	4.84	-0.21	1.30	wPt-9798~wPt-9133	4.41	0.45	10.36	EAW74	wPt-9133	4.63	0.22	1.79			
3R	35.2					rPt-507396	2.55	-0.28	2.45	EAW78					rPt-507396	-0.33	0.9
4R	65.4	rPt-410866	5.00	-0.19	1.14	rPt-401323	2.68	-0.24	7.09	DH07	rPt-410866	3.70	-0.18	1.26	rPt-410866	-0.22	1.9
5R	18.9	rPt-399681	44.93	0.98	32.70	rPt-399681	27.27	1.02	35.02	EAW78	rPt-399681	38.78	0.98	40.27	rPt-399681	1.04	17.4
5R	35.2	rPt-402367	3.42	0.26	2.22										rPt-402367	0.30	1.8
6R	46.2	rPt-401125	5.97	-0.24	1.60	rPt-398551	2.61	1.28	13.86	DH06					rPt-401125	-0.24	1.4
7R	40.4					rPt-410852	2.77	-0.32	2.95	EAW78					rPt-400878	0.21	1.1

Table S8 | Probabilities in the paired *t* test for average statistical power and mean absolute deviation (MAD) among genome-wide composite interval mapping (CIM), CIM and empirical Bayes

Case	Genome-wide CIM (GCIM) & CIM		Empirical Bayes & CIM		Genome-wide CIM & empirical Bayes	
	**Power**	**MAD**	**Power**	**MAD**	**Power**	**MAD**
**Monte Carlo simulation experiment I (20 main-effect QTL were simulated)**						
20 main-effect QTL	2.09E-4	0.0253	1.60E-4	0.0162	0.4790	0.0196
Small effect QTL	0.0178	0.0115	0.0173	0.0102	0.7177	6.84E-4
Closely linked QTL	0.0049	0.1121	0.0039	0.0773	0.5813	0.0247
**Monte Carlo simulation experiment I (20 main-effect QTL along with polygenic background were simulated)**						
20 main-effect QTL	1.27E-4	0.0576	1.83E-4	0.0317	0.5237	0.0164
Small effect QTL	0.0147	0.0034	0.0253	0.0053	0.3955	0.5456
Closely linked QTL	0.0039	0.1930	0.0031	0.1205	0.1846	0.0182
**Monte Carlo simulation experiment I (20 main-effect QTL along with epistatic background were simulated)**						
20 main-effect QTL	0.0056	0.0249	0.0035	0.0220	0.6612	0.0322
Small effect QTL	0.4219	0.0024	0.5909	0.0026	0.0997	0.5707
Closely linked QTL	0.0326	0.1370	0.0167	0.1070	0.5362	0.0272

Table S9 | QTL mapping derived from inclusive composite interval mapping (ICIM) in the first to third Monte Carlo simulation experiments (200 replicates)

QTL	The first simulation experiment			The second simulation experiment			The third simulation experiment		
	Power (%)	Effect (SD,MSE,MAD)	Position (SD,MSE)	Power (%)	Effect (SD,MSE,MAD)	Position (SD,MSE)	Power (%)	Effect (SD,MSE,MAD)	Position (SD,MSE)
1	100	4.47(0.17,0.03,0.14)	50.02(0.26,0.07)	100	4.45(0.36,0.13,0.29)	50.00(0.33,0.11)	100	4.49(0.31,0.10,0.24)	50.02(0.50,0.25)
2	100	3.15(0.21,0.04,0.16)	125.03(0.54,0.29)	100	3.14(0.33,0.11,0.27)	124.96(0.62,0.39)	99.5	3.16(0.32,0.10,0.25)	125.01(0.98,0.95)
3	99.5	-2.26(0.22,0.05,0.17)	199.95(0.74,0.54)	99.5	-2.23(0.33,0.11,0.26)	200.09(1.22,1.48)	99	-2.32(0.38,0.15,0.30)	200.20(1.37,1.90)
4	98	-1.57(0.21,0.05,0.16)	235.01(1.26,1.58)	94.5	-1.66(0.32,0.11,0.25)	235.08(1.14,1.30)	85	-1.63(0.32,0.11,0.26)	234.78(1.91,3.67)
5	99.5	2.24(0.30,0.09,0.24)	349.92(0.74,0.55)	99	2.29(0.42,0.18,0.35)	349.98(1.06,1.11)	86.5	2.46(0.73,0.58,0.46)	350.02(1.23,1.51)
6	100	3.20(0.33,0.11,0.25)	360.01(0.51,0.26)	99.5	3.25(0.55,0.31,0.39)	359.99(0.62,0.38)	95.5	3.43(0.77,0.65,0.57)	359.83(1.07,1.17)
7	20	1.26(0.24,0.08,0.24)	609.93(1.37,1.83)	25	1.23(0.35,0.13,0.30)	609.72(1.39,1.96)	5	1.33(0.44,0.23,0.34)	609.9(2.51,5.7)
8	25	-1.19(0.29,0.09,0.26)	630.10(1.69,2.82)	23	-1.27(0.38,0.17,0.34)	630.79(5.45,29.66)	4	-1.40(0.35,0.20,0.39)	629.38(0.92,1.13)
9	69	0.83(0.14,0.02,0.13)	800.05(2.20,4.79)	37.5	1.00(0.28,0.13,0.27)	799.95(2.12,4.43)	25.5	1.06(0.21,0.13,0.29)	799.98(2.35,5.43)
10	100	1.75(0.25,0.06,0.20)	889.89(1.10,1.23)	92	1.80(0.34,0.12,0.28)	889.87(1.31,1.72)	78.5	1.86(0.36,0.15,0.31)	889.92(1.53,2.33)
11	100	3.84(0.32,0.11,0.25)	905.01(0.34,0.12)	100	3.87(0.49,0.24,0.37)	904.97(0.43,0.19)	100	4.10(0.73,0.62,0.58)	904.96(0.74,0.54)
12	99.5	2.23(0.26,0.07,0.21)	920.05(0.63,0.40)	100	2.23(0.38,0.14,0.30)	919.94(0.98,0.96)	97	2.23(0.44,0.19,0.36)	920.04(1.34,1.79)
13	93.5	-1.33(0.17,0.03,0.14)	1099.93(1.28,1.63)	85	-1.33(0.30,0.09,0.24)	1100.07(2.05,4.19)	77	-1.41(0.31,0.10,0.24)	1100.05(1.88,3.51)
14	88	-1.02(0.19,0.03,0.15)	1210.00(1.91,3.64)	66	1.14(0.30,0.11,0.26)	1210.08(1.96,3.81)	47	-1.19(0.26,0.10,0.23)	1209.88(2.52,6.29)
15	99.5	-2.24(0.24,0.06,0.18)	1304.93(0.81,0.66)	99	-2.23(0.35,0.12,0.28)	1304.92(1.05,1.10)	92.5	-2.28(0.45,0.20,0.35)	1304.94(1.31,1.71)
16	91	1.95(0.45,0.34,0.50)	1335.55(1.31,2.01)	83.5	2.09(0.46,0.47,0.58)	1335.54(1.43,2.32)	74.5	2.14(0.42,0.49,0.61)	1335.45(1.76,3.28)
17	40.5	1.44(0.36,0.33,0.44)	1345.01(1.50,2.23)	25.5	1.75(0.50,0.81,0.75)	1344.47(1.92,3.90)	9	2.22(0.48,1.70,1.22)	1344.78(2.21,4.67)
18	96.5	-1.61(0.30,0.11,0.27)	1365.29(1.23,1.60)	89	-1.62(0.38,0.16,0.32)	1365.31(1.35,1.92)	67	-1.49(0.33,0.17,0.36)	1365.69(1.60,3.01)
19	66	0.82(0.21,0.05,0.15)	1799.85(3.02,9.07)	44	0.98(0.28,0.15,0.28)	1799.97(2.20,4.81)	22	1.04(0.17,0.13,0.33)	1799.84(2.71,7.20)
20	82	0.93(0.16,0.03,0.13)	2300.18(1.82,3.34)	57	1.04(0.26,0.09,0.23)	2299.98(2.11,4.42)	40.5	1.13(0.23,0.11,0.25)	2300.38(2.44,6.01)

QTL parameters in the three Monte Carlo simulation experiments were shown in Tables S1, S2 and S3, respectively.

Simulated Datasets

Simulated_datasets.zip is a *.zip file that includes simulated_datasets.csv. In the simulated experiment I, the phenotypic values are in the first column and the genotypic values for all the 481 markers are in the fourth to 484th columns. In the genotypic datasets in backcross population, 1 is AA marker genotype and -1 is Aa marker genotype. In the simulated experiment II, the phenotypic values are in the second column and the genotypic values for all the 481 markers are also in the fourth to 484th columns. In the simulated experiment III, the phenotypic values are in the third column and the genotypic values for all the 481 markers are also in the fourth to 484th columns. In other words, the genotypic datasets in the second and third simulation experiments were same as those in the first simulation experiment. In the simulated_datasets.csv, each of 1 to 80000 rows indicates one individual, each of 4 to 484 columns represents one marker and 400 individuals consist of one sample. The *i*th sample consists of the $[(i-1)\times 400+1]$th row to $(400i)$th row ($i=1,\cdots,200$).
